# Editorial: Hot Topics in Cellular Neuropathology

**DOI:** 10.3389/fncel.2022.895861

**Published:** 2022-04-19

**Authors:** Dirk M. Hermann, Aurel Popa-Wagner, Luca Peruzzotti-Jametti, Matthias Gunzer

**Affiliations:** ^1^Department of Neurology, University Hospital Essen, Essen, Germany; ^2^Experimental Research Center in Normal and Pathological Aging, University of Medicine and Pharmacy, Craiova, Romania; ^3^Department of Clinical Neurosciences, University of Cambridge, Cambridge, United Kingdom; ^4^Institute of Experimental Immunology and Imaging, University Hospital Essen, Essen, Germany

**Keywords:** neurodegeneration, neuroinflammation, microglia, cognitive deficits, dementia

Representing a particularly vulnerable tissue, the brain is surrounded by the blood-brain barrier (BBB), which efficiently prevents the entry of harmful blood-derived toxins, microbes, and inflammatory signals. Formed by tight junctions and adherens junctions that impede paracellular leakage, the BBB limits the access to the central nervous system (CNS) more effectively than barriers in other organs (Hermann and ElAli, 2012; Schaeffer and Iadecola, 2021). The cerebral microvascular endothelium itself exhibits very little vesicular transcytosis, which further strengthens BBB tightness. In addition, endothelial transporters on the luminal and abluminal endothelial membranes actively remove toxic and proinflammatory molecules from the CNS (Hermann et al., 2006), whereas adhesion molecules for blood-derived leukocytes are expressed at low levels under physiological conditions (Dietrich, 2002; Iadecola and Anrather, 2011). As a consequence of all these features, the CNS is considered a highly immunoprivileged tissue.

Inflammation, involving innate and adaptive immune responses, compromises the BBB's integrity and consequently cerebral function (Iadecola and Anrather, 2011; Grüneboom et al., 2022). In acute diseases (such as infections, brain ischemia, or trauma), inflammation promotes acute neurodegenerative processes (Iadecola and Anrather, 2011) and sustained inflammation is thought to be a major factor responsible for cognitive decline. In chronic disorders (including neurodegenerative and neuropsychiatric disorders), a subtle and persistent grade of neuroinflammation induces long-lasting neurodegenerative sequelae that ultimately compromise brain functioning (Peruzzotti-Jametti et al., 2021).

The Hot Topics in Cellular Neuropathology Research Topic gathered a selection of noteworthy papers, which explore the link between BBB function, persistent immune cell activation, and consequent CNS damage after acute (infectious) and chronic (neuropsychiatric) disorders.

In their review, Bohmwald et al. examined the long-term consequences of viral encephalitis, analyzing how neurotropic viruses reach the brain (via paracellular or transcellular mechanisms) to infect neurons and brain resident glial cells, which in turn secrete proinflammatory cytokines and chemokines that attract blood-derived immune cells. For some viruses (such as arboviruses, enteroviruses, herpesviruses, retroviruses or orthomyxoviruses), the brain represents the principal infection target, whereas for others (such as orthomyxoviruses, orthopneumoviruses or coronaviruses), the brain is a secondary target site. Following an early innate immune infiltrate characterized by neutrophils and macrophages, increasing numbers of CD8^+^ and CD4^+^ T cells accumulate in the brain tissue in the chronic disease phase, persisting even after the initial viral infection has subsided. The authors discuss how this persistent immune activation contributes to long-term neuropsychiatric disorders, cognitive impairment, motor dysfunctions, and neurodegenerative states *via* mechanisms including microglial overactivation, synaptic degeneration, neuronal apoptosis, and reactive astrogliosis.

Coronaviruses have gained prominence due the recent COVID-19 pandemic. Although significant neurological deficits have been associated with SARS-CoV-2 infection, their pathogenesis remains unclear. Interestingly, while only low (or undetectable) levels of SARS-CoV-2 have been reported in human post mortem brain specimens, the S1 spike protein located on the surface of SARS-CoV-2 can be released from viral membranes and cross the BBB and may cause CNS damage (Matschke et al., 2020; Meinhardt et al., 2021; Song et al., 2021). Therefore, in their original manuscript Datta et al. investigated the hypothesis that SARS-CoV-2 S1 protein can directly induce neuronal injury. In live cell imaging studies, incubation of primary human cortical neurons and primary mouse hippocampal neurons with SARS-CoV-2 S1 protein resulted in the accumulation of the S1 protein in endolysosomes, as well as in endolysosome de-acidification. Further, SARS-CoV-2 S1 protein induced aberrant endolysosome morphology and neuritic varicosities. These data are interpreted that SARS-CoV-2 S1 protein can directly induce neuritic dystrophy, which could contribute to the high incidence of neurological deficits associated with COVID-19. These data propose a link between SARS-CoV-2 and CNS integrity. However, the mechanism via which SARS-CoV-2 S1 protein induces neuronal injury will have to be further scrutinized, and *in vivo* studies are still needed to assess the true relevance of endolysosomal S1 spike protein accumulation for neuritic dystrophy and brain pathology.

With the COVID-19 pandemic, interest has also re-emerged on the mechanisms by which an initial viral infection can cause long lasting neuropsychiatric symptoms. Indeed, excessive exhaustion, pain, cognitive deficits, and sleep abnormalities often persists over weeks and months following SARS-CoV-2 infection, for which the term *long-COVID syndrome* was coined. In a Hypothesis and Theory paper, Sfera et al. proposed that COVID-19-upregulated angiotensin-II triggers premature endothelial cell senescence, disrupting the intestinal and blood-brain barriers. The authors hypothesize that post-viral sequelae, including myalgic encephalomyelitis (ME) and chronic fatigue syndrome (CFS), might be promoted by gut microbe or toxin translocation from the gastrointestinal tract into other tissues, including the brain. As evidence strengthening their hypothesis, the authors discuss data demonstrating the SARS-CoV-2 interaction with proteins encountered in the host, such ACE2 and bacterial lipopolysaccharide, facilitates vascular endothelial senescence and intestinal epithelial cell disruption via mechanisms that include transforming growth factor-β (TGFβ), high-mobility group protein-B1 (HMGB1), and a disintegrin and metalloprotease-17 (ADAM17) signaling. The authors suggest that targeting microbial translocation and cellular senescence may help to ameliorate the neuropsychiatric symptoms of SARS-CoV-2 infection.

Among neuropsychiatric disorders, depression is the most common and widespread in our society. For many years major depressive disorder has been associated with subtle brain inflammatory responses, while subtle neuroinflammation has also been found in the brains of suicidal subjects (Hoyo-Becerra et al., 2014). In their review, Goncalves de Andrade et al. evaluated the role of microglia as a hub for suicide neuropathology. The authors summarize *in vivo* translocator protein (TSPO) positron emission tomography (PET) evidence suggesting microglial overactivation in the brains of depressed subjects exhibiting suicidal ideation, compared to subjects without suicidal thoughts. They then postulate that microglial overactivation facilitates the brain access of peripheral leukocytes, including monocytes and neutrophils, into the brain by release of CC-chemokine ligand-2 (CCL2). Microglia in suicide victims has been described to exhibit distinct morphological features, namely an increased volume, ellipsoid soma and reduced higher order branches, indicative of a depression-associated priming that might reflect a state of elevated stress susceptibility. These changes are possible responses to hypothalamic pituitary axis (HPA) dys function, disturbed tryptophan metabolism, and oxidative stress. Specifically, oxidative stress might predispose microglia to dystrophic degenerative changes resembling those of *dark microglia*, which the authors had characterized before in chronic stress and neurodegenerative conditions (Bisht et al., 2016). Dark microglia have disrupted mitochondria, dilation of the Golgi apparatus and endoplasmic reticulum, abundant endosomes, and cell shrinkage. Dark microglia appear to be much more active than the normal microglia, reaching out for synaptic clefts, and encircling axon terminals and dendritic spines with their highly ramified and thin processes. They stain for the myeloid cell marker ionized calcium binding adaptor molecule-1 (Iba-1), and strongly express CD11b and microglia-specific 4D4 in their processes encircling synapses, and express triggering receptor expressed on myeloid cells (TREM2) when they get exposed to amyloid plaques (Bisht et al., 2016). The authors suggest that microglia (and specifically dark microglia) may participate in mechanisms that control suicide resistance and susceptibility. Therefore, the selective modulation of processes that boost microglial resistance and homeostasis might be a new target to treat depression and ultimately prevent suicide deaths.

The papers of this Research Topic share the view that subtle, unbalanced neuroinflammation persisting in the chronic phases of brain injury may unfavorably influence brain integrity and function, similarly to the subtle neuroinflammation associated with psychosocial stress or major depressive disorders. The similarities of pathophysiological processes in the above studies are intriguing regarding the diversity of disease states, prompting the question that the prevention of inflammatory processes might offer huge potential for stabilizing physical, cognitive and mental health. Further Hot Topics sections within this journal are warranted aiming to identify studies that have impact in the field.

## Author Contributions

DMH drafted the text. All authors revised and finalized it. All authors contributed to the article and approved the submitted version.

## Funding

Supported by the German Research Foundation (grants 389030878 and 405358801 to DMH and MG) and the Wellcome Trust (CRCD Fellowship, RG G105713 to LP-J).

## Conflict of Interest

The authors declare that the research was conducted in the absence of any commercial or financial relationships that could be construed as a potential conflict of interest.

## Publisher's Note

All claims expressed in this article are solely those of the authors and do not necessarily represent those of their affiliated organizations, or those of the publisher, the editors and the reviewers. Any product that may be evaluated in this article, or claim that may be made by its manufacturer, is not guaranteed or endorsed by the publisher.
